# Machine-learning derived identification of prognostic signature to forecast head and neck squamous cell carcinoma prognosis and drug response

**DOI:** 10.3389/fimmu.2024.1469895

**Published:** 2024-12-19

**Authors:** Sha-Zhou Li, Hai-Ying Sun, Yuan Tian, Liu-Qing Zhou, Tao Zhou

**Affiliations:** ^1^ Department of Otorhinolaryngology, Union Hospital, Tongji Medical College, Huazhong University of Science and Technology, Wuhan, Hubei, China; ^2^ Department of Geriatrics, Union Hospital, Tongji Medical College, Huazhong University of Science and Technology, Wuhan, Hubei, China

**Keywords:** machine learning, HNSCC, DEGs, tumor microenvironment, immunotherapy

## Abstract

**Introduction:**

Head and neck squamous cell carcinoma (HNSCC), a highly heterogeneous malignancy is often associated with unfavorable prognosis. Due to its unique anatomical position and the absence of effective early inspection methods, surgical intervention alone is frequently inadequate for achieving complete remission. Therefore, the identification of reliable biomarker is crucial to enhance the accuracy of screening and treatment strategies for HNSCC.

**Method:**

To develop and identify a machine learning-derived prognostic model (MLDPM) for HNSCC, ten machine learning algorithms, namely CoxBoost, elastic network (Enet), generalized boosted regression modeling (GBM), Lasso, Ridge, partial least squares regression for Cox (plsRcox), random survival forest (RSF), stepwise Cox, supervised principal components (SuperPC), and survival support vector machine (survival-SVM), along with 81 algorithm combinations were utilized. Time-dependent receiver operating characteristics (ROC) curves and Kaplan-Meier analysis can effectively assess the model’s predictive performance. Validation was performed through a nomogram, calibration curves, univariate and multivariate Cox analysis. Further analyses included immunological profiling and gene set enrichment analyses (GSEA). Additionally, the prediction of 50% inhibitory concentration (IC50) of potential drugs between groups was determined.

**Results:**

From analyses in the HNSCC tissues and normal tissues, we found 536 differentially expressed genes (DEGs). Subsequent univariate-cox regression analysis narrowed this list to 18 genes. A robust risk model, outperforming other clinical signatures, was then constructed using machine learning techniques. The MLDPM indicated that high-risk scores showed a greater propensity for immune escape and reduced survival rates. Dasatinib and 7 medicine showed the superior sensitivity to the high-risk NHSCC, which had potential to the clinical.

**Conclusions:**

The construction of MLDPM effectively eliminated artificial bias by utilizing 101 algorithm combinations. This model demonstrated high accuracy in predicting HNSCC outcomes and has the potential to identify novel therapeutic targets for HNSCC patients, thus offering significant advancements in personalized treatment strategies.

## Introduction

HNSCC is the sixth common cancer globally. The incidence of NHSCC continues to rise, with projections estimating 1.08 million cases by 2030 (1, 2). Although there are advancements in multimodality treatment, the five-year survival rate remains below 50% (3, 4). However, the heterogeneity of HNSCC, arising from diverse etiologies and underlying molecular alterations, presents significant challenges in tailoring precise treatments. This heterogeneity can lead to both over-treatment and under-treatment of patients (5).

Immunotherapy was a promising treatment modality for HNSCC (6). But there was still a problem that only a few drugs were used to treatment in this domain such as EGFR targeting monoclonal antibody, anti-programmed death-1 (PD-1) inhibitors and PD-L1 (5, 7). Most of them are aimed to recurrent or metastatic HNSCC. And less than 20% of immune checkpoint inhibitors can have effects in their patient (8, 9). Thus potential targets is urgently needed for HNSCC immunotherapy in different stage.

In this study, we selected 18 remarkably prognostic genes and constructed a prognostic model by 101 combinational algorithms. Using this model, HNSCC patients were divided into two groups based on their risk scores. It is expected to help doctors to predict survival time of patients, choose better treatment strategies, provide new gene targets for immunotherapy and apply more sensitive drugs to patients between two groups. This study has significant benefits in terms of further immune research, precision treatment and improvements of clinical outcomes.

## Method

### Collection of databases

The clinical characteristics, expression profiles and relevant data for NHSCCs were obtained from The Cancer Genome Atlas (TCGA) and Gene Expression Omnibus (GEO) datasets. Our study included 825 samples from two cohorts: TCGA-HNSC (n = 555) and GSE65858 (n = 270). For each patient, RNA-sequencing and other clinical information were collected. Detailed information is provided in **Supplementary Table 1**. RNA-sequencing data underwent log-2 fold-change (log-2FC). Preprocessed of the data was conducted using the robust multi-array averaging (RMA) algorithm, implemented in the “affy” package. This preprocessing step included background correction, normalization, and summarization to ensure the data were suitable for downstream analyses.

### Construction of MLDPM and Kaplan-Meier survival analysis

To identify DEGs, we utilized the caret R package (R version 4.4.0) and Strawberry Perl to contrast the TCGA and GEO databases, resulting in the identification of 536 DEGs. Univariate Cox regression analysis was used to analyze these DEGs, yielding 18 risk genes that were subsequently incorporated into the risk model.

To construct a robust risk model, the expression profiles of the prognostic gene were transformed into z-scores to enhance comparability across different samples. The procedure was followed to generate signatures:

a) 10 machine-learning algorithms were integrated, including random survival forest (RSF), Ridge, Lasso, generalized boosted regression modeling (GBM), supervised principal components (SuperPC), CoxBoost, partial least squares regression for Cox (plsRcox), elastic network (Enet), Stepwise Cox and survival support vector machine (Survival-SVM). These algorithms were combined using 10-fold cross-validation approaches in a random manner, producing a total of 101 combinational algorithms tailored for enhanced predictive accuracy (10–12). Detailed descriptions of each algorithm were outlined in previous research by Hu et al (11, 13).
b) For every combinational algorithm, data from TCGA and GEO were respectively used to construct and validate the prognostic model. The first algorithm was applied to select variables from 18 selected prognostic genes, and the last was utilized construct the risk model based on variables.
c) For ensuring the most predictive and reliable model was selected, average concordance index (C-index) was calculated across TCGA-HNSC and GSE65858 cohorts to select the greatest combinational algorithm.

### Validation of the MLDPM

Univariate and multivariate Cox proportional hazard regression analyses were carried out to validate the independent predictive significance of clinical features for OS. ROC curve analysis was conducted using the “timeROC” R package to evaluate the predictive ability of the HNSCC prognostic model and other clinicopathological signatures. Furthermore, the C-index of the MLDPM was compared with that of other clinical factors to assess the superiority of our prognostic model. The C-index represents the discriminatory ability of the model, where a higher value indicates better predictive performance.

### Nomogram and calibration

The rms R package was employed to construct a nomogram for predicting the 1-, 3-, and 5-year OS. A nomogram provided a graphical representation of the predictive model, assigning points to each variable, with the total points correspond to the predicted outcome probability.

To evaluate the nomogram’s performance, calibration curves were generated to compare the predicted probabilities with the actual outcomes. These curves demonstrated the consistency between the predicted and observed results.

### Estimate and enrichment analysis

The tumor estimate scores (TME) were calculated by the estimate R package. The tumor mutational burden (TMB) is associated with the formation of neoantigens that can trigger an immune response against tumors. The TMB was compared to reflect the diverse immune environment in two groups. Immunotherapeutic prediction was performed by calculating the Tumor Immune Dysfunction and Exclusion (TIDE) prediction score. The score was then employed to evaluate the degree of immunotherapy responsiveness similarity among patients with HNSCC. Potential molecular mechanisms were identified through GSEA. Following the DEGs analysis, all genes were ranked based on log-2FC. To uncover the potential biological functions and signaling pathways involved, Gene Ontology (GO) classification and Kyoto Encyclopedia of Genes and Genomes (KEGG) enrichment analysis were executed using the “clusterProfiler” tool. The top five significant biological pathways were then chosen for graphical representation.

### The tumor immune microenvironment (TIME) and immune subtypes

The analysis of immune cell factors in two risk groups, based on GSEA results, was conducted using software such as TIMER, XCELL, CIBERSORT, EPIC, MCPcounter, QUANTISEQ, and CIBERSORT on TIMER 2.0. This comprehensive approach provided detailed insights into the immune cell infiltration profiles.

The immune cell infiltration status within the HNSCC patient population was quantified, and the tumor infiltration estimation data derived from TCGA were retrieved from the appropriate online platform for further analysis. Differences in immune infiltrating cells between the two risk groups were analyzed using Wilcoxon signed-rank test. Visualization of the results was done using the “scales,” “limma,” “ggtext,” and “ggplot2” R packages, with a bubble chart representation.

Additionally, the analysis of immune checkpoint activation and TIME scores between the risk groups was carried out using the “ggpubr” R package to compare and visualize the differences.

### Checkpoints and prediction of potential drugs

With the help of the “oncoPredict” package, the sensitivity analysis of several drugs using the Genomics of Drug Sensitivity in Cancer (GDSC) database was conducted. This analysis focused on evaluating the response of cancer cells to different drugs based on their genomic profiles and drug sensitivity data. Furthermore, the IC50 difference between groups was explored through a differential expression analysis. It revealed the significant differences associated with the risk score in drug sensitivity.

### Statistical analysis

Statistical data analysis in this study was conducted by Bioconductor packages in R 4.4.0. HNSCC patients’ predictive ability of prognostic signatures and clinicopathological characteristics were assessed through the ROC curve, with the “timeROC” R package being employed for this analysis. Univariate and multivariate Cox analyses were performed to determine the independent prognostic value of OS clinical characteristics in HNSCC patients. The Kaplan-Meier method was used to evaluate survival prediction in the patient cohort. The prediction for OS was showed in the nomogram. Immune cell, immune function, subtypes, estimate scores, TMB, and TIDE drawn an integrated picture of tumor microenvironment. Besides, GSEA enrichment explained it in a microcosmic viewpoint. Lastly drug sensitivity analysis would demonstrated a way to cure or stop the progression of the tumor.

## Result

### MLDPM construction and validation

**Figure 1:**
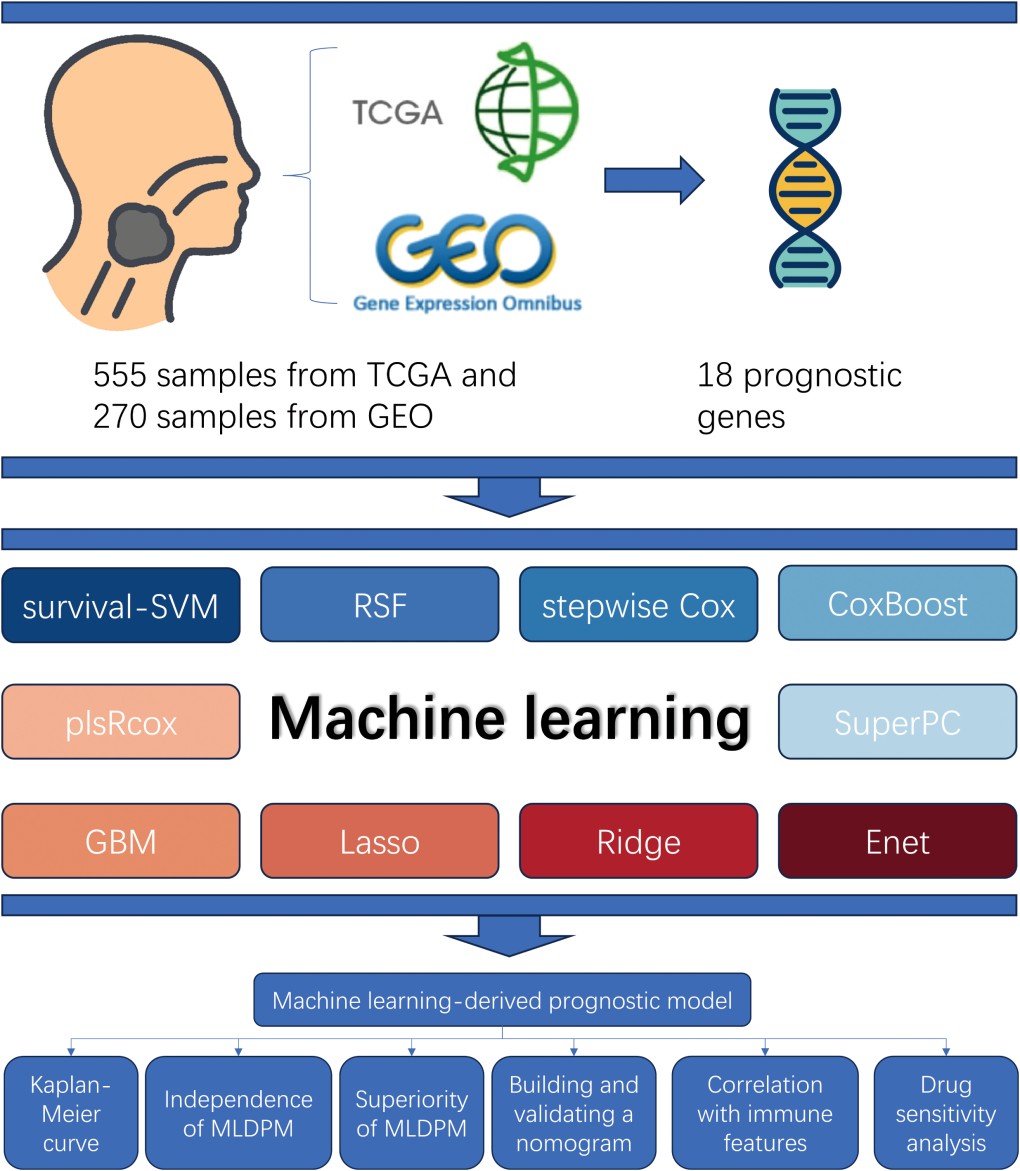
Study design overview. The samples from TCGA and GEO (total n = 825) were used to obtain 18 prognostic genes matrix expression data. The MLDPM was constructed through 101 combinational algorithms consisted of 10 machine-learning algorithms. Subsequently, Kaplan-Meier analysis and univariate/multivariate Cox analyses were applied to assess its prediction ability, and the model was verified by a nomogram and calibration curves. Furthermore, immune analysis, and GSEA were performed, and the IC50 prediction in the risk groups was calculated.

**Figure 2:**
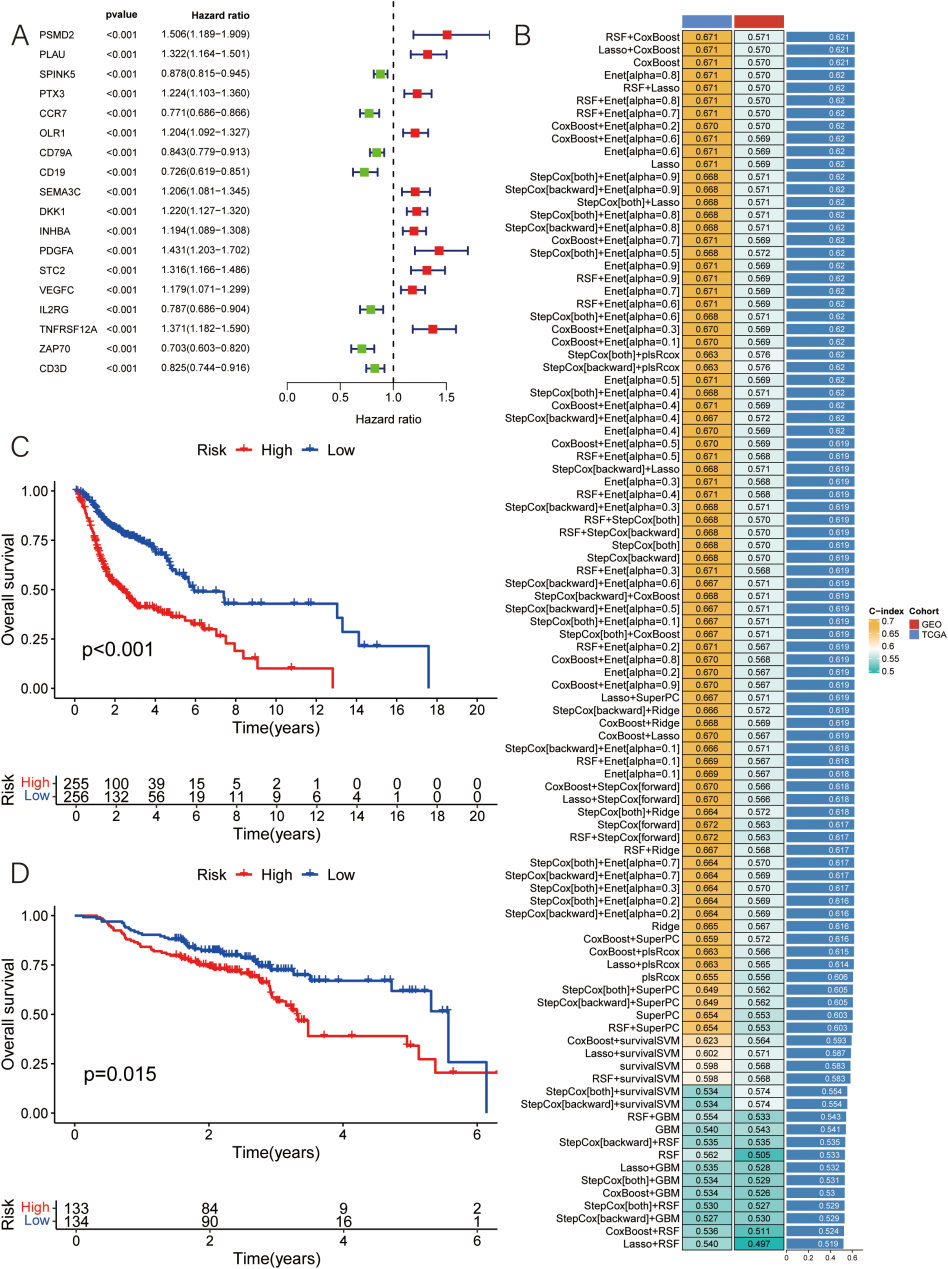
Construction of MLDPM. **(A)** 101 algorithm combinations were used to identify the MLDPM. The average C-index of two cohorts (TCGA-HNSC and GSE65858) was calculated. **(B)** The forest plot exhibited the results of the univariate-cox regression analysis. **(C)** Overall survival in the different risk groups of TCGA. **(D)** Overall survival in the different risk groups of GEO.

**Figure 1** shows the prognostic model construction process. Initially, we obtained 59427 genes and 555 HNSCC symbols from the TCGA database. By comparing the 555 TCGA symbols with 270 symbols from the GEO database, we identified a total of 536 DEGs. 536 candidate genes are screened by univariate Cox regression analysis to identify significant prognostic risk-related genes, with a threshold p-value less than 0.001. This analysis led to the selection of 18 prognostic genes, including 11 upregulated genes and 7 downregulated genes (**Figure 2A**).

Subsequently, based on the expression levels of these 18 selected features, along with survival time and status as input data, a total of 101 algorithm patterns were applied using 10 machine learning methods on the TCGA-HNSC cohort as the training set and the GSE65858 cohort as the test set (**Figure 2B**). By assessing the average C-index of the different algorithm patterns, the RSF+CoxBoost combination was identified as having the highest average C-index of 0.621, indicating its effectiveness in predicting patient outcomes.

This RSF+CoxBoost model facilitated the identification of key prognostic features among the 18 selected genes. Using the median risk value, the HNSCC samples were classified into two groups, the low-risk and the high-risk group. Kaplan-Meier survival analyses demonstrated that the survival outcomes in HNSCC is intimately connected with the risk score, with low-risk patients showing higher overall survival rates (**Figures 2C, D**).

### Verification of the MLDPM accuracy

**Figure 3:**
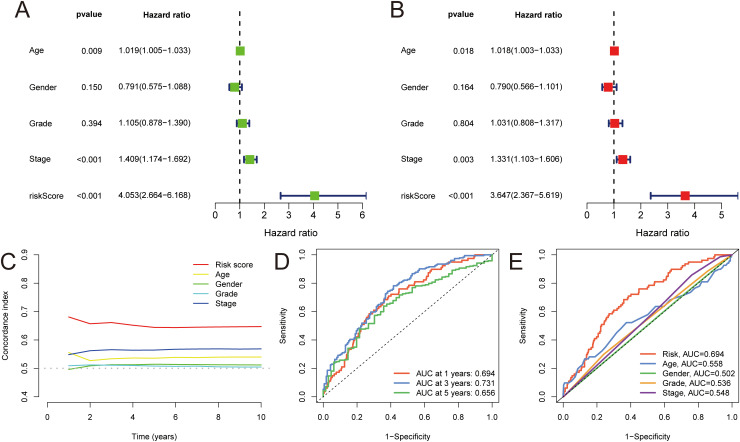
Validation of MLDPM. **(A)** Univariate-cox regression analysis and **(B)** Multivariate-cox regression analysis indicated the MLDPM as an independent risk factor for HNSCC combined with other clinical features. **(C)** C-index of the MLDPM and clinical factors for evaluating treatment outcome. **(D)** Time-ROC analysis for predicting prognosis. **(E)** ROC analysis of the risk score and clinically relevant pathological factors.

Univariate and multivariate Cox regression analysis were carried out to assess the prognostic significance of the MLDPM (HRs were 4.053, 95% CI: 2.664–6,168, *p*< 0.001 and 3.647, 95% CI: 2.367–5.619, *p*< 0.001, respectively), incorporating immune-related genes, age, and stage (**Figures 3A, B**). These analyses revealed that the MLDPM indeed had a significant predictive value for NHSCC patients, suggesting its association with their risk profile. The risk score consistently exhibited a higher concordance index compared to individual clinical components over time. This finding suggests that the overall risk grade based on the MLDPM is a more reliable indicator for predicting the prognosis, potentially outperforming traditional clinical variables (**Figure 3C**). AUC values of 1-, 3-, and 5-year survival outcomes were 0.694, 0.731, and 0.656, respectively (**Figure 3D**). Furthermore, for the risk grade, determined by the MLDPM, the value surpassed those of other clinicopathological factors like age, gender, stage, and grade. This result highlights the robustness and reliability of the MLDPM in terms of differentiating HNSCC patient outcomes, as it provides a more discriminative tool for prognosis prediction compared to these conventional parameters (**Figure 3E**).

### Prognostic nomogram development and evaluation

**Figure 4:**
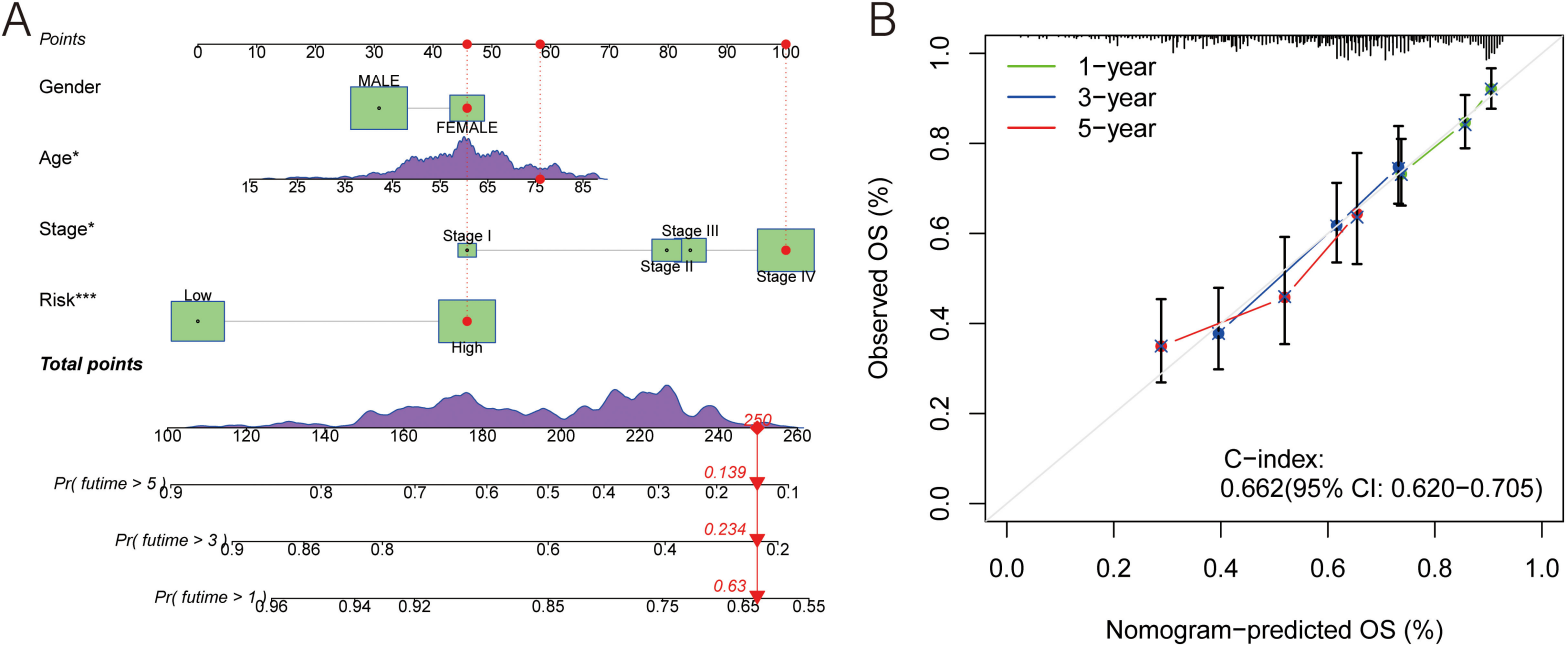
A prognostic nomogram. **(A)** Nomogram model presenting the MLDPM and clinicopathological factors. **(B)** Nomogram model predicting overall survival using calibration curves.

In this study, a nomogram was developed for individuals with HNSCC based on gender, age, stage, and risk score. Doctors could roughly predict patients’ OS using their clinical information (**Figure 4A**). Additionally, the calibration curves demonstrated a high C-index (0.658,95% CI: 0.615-0.701), indicating the superior performance of the nomogram (**Figure 4B**). This suggests that the personalized OS prediction model incorporating these clinical and molecular variables could be a valuable tool for prognostic assessment in HNSCC patients.

### Tumor immune microenvironment analysis

**Figure 5:**
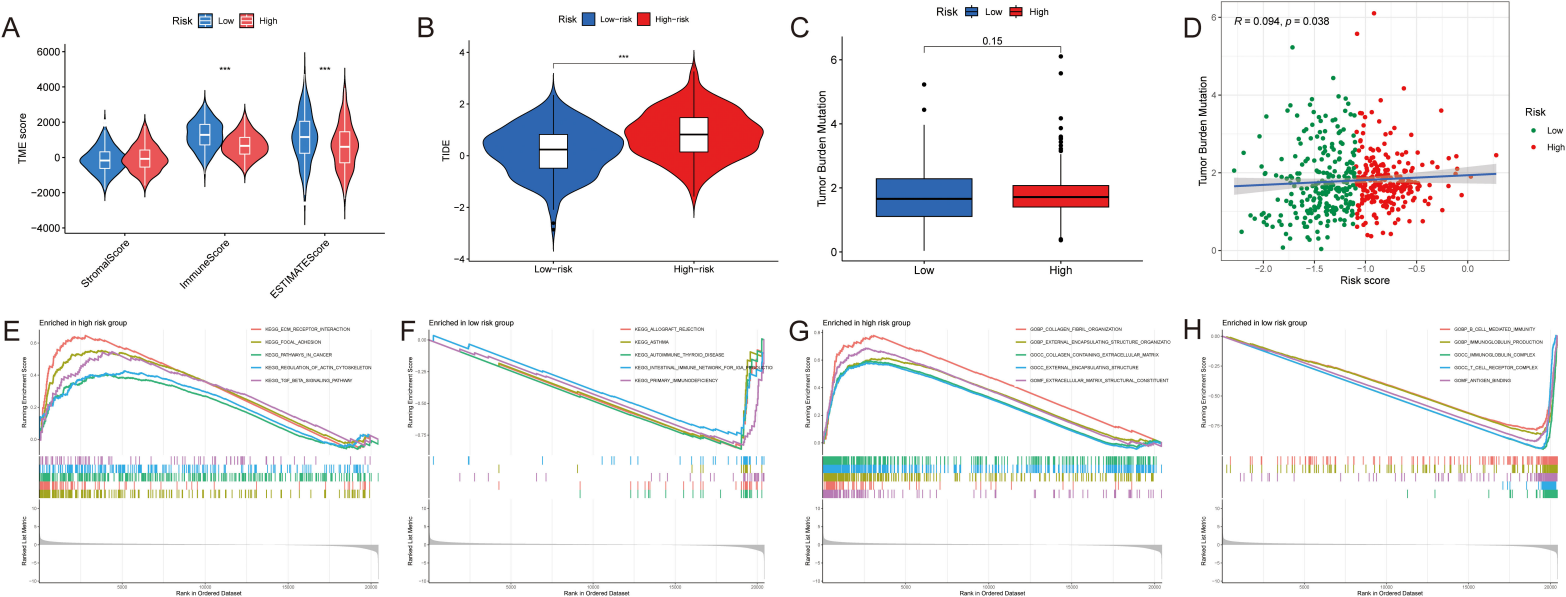
Tumor components analyses of HNSCC. **(A–C)** The difference of the **(A)** TME score, **(B)** TIDE and **(C)** TMB between two groups. **(D)** The correlation between the TMB and the risk score. **(E, F)** GSEA based on the KEGG analysis in two groups. **(G, H)** GSEA based on the GO analysis in two groups. ***p < 0.001.

The immune scores in the low-risk group were significantly higher (p< 0.001), and it was the immune factors that played a role of the main character (p< 0.001) (**Figure 5A**). The results of TIDE can illustrate this from another side (**Figure 5B**). The TMB scores were computed according to the TCGA somatic mutation data. It was positively associated with high-risk scores, but the difference between groups was not big enough (**Figures 5C, D**). Above all, the difference between two groups was mainly due to their immune function in the TIME. Thus, we needed to analyze it in more details.

Initially, we used GSEA enrichment analysis to select the top 5 ways of two groups. Several pathways like intestinal immune network for IgA production and primary immunodeficiency were associated with the low-risk group, while ECM-receptor interaction, focal adhesion, pathways in cancer and TGF-β signaling pathway were found in the high-risk group (**Figures 5E, F**). And compared with high expression of collagen fibril organization, extracellular matrix structural constituent in the high-risk group, B cell mediated immunity, antigen binding, T cell receptor complex as well as immunoglobulin complex produced more in the low-risk group (**Figures 5G, H**).

### Cell analysis

**Figure 6:**
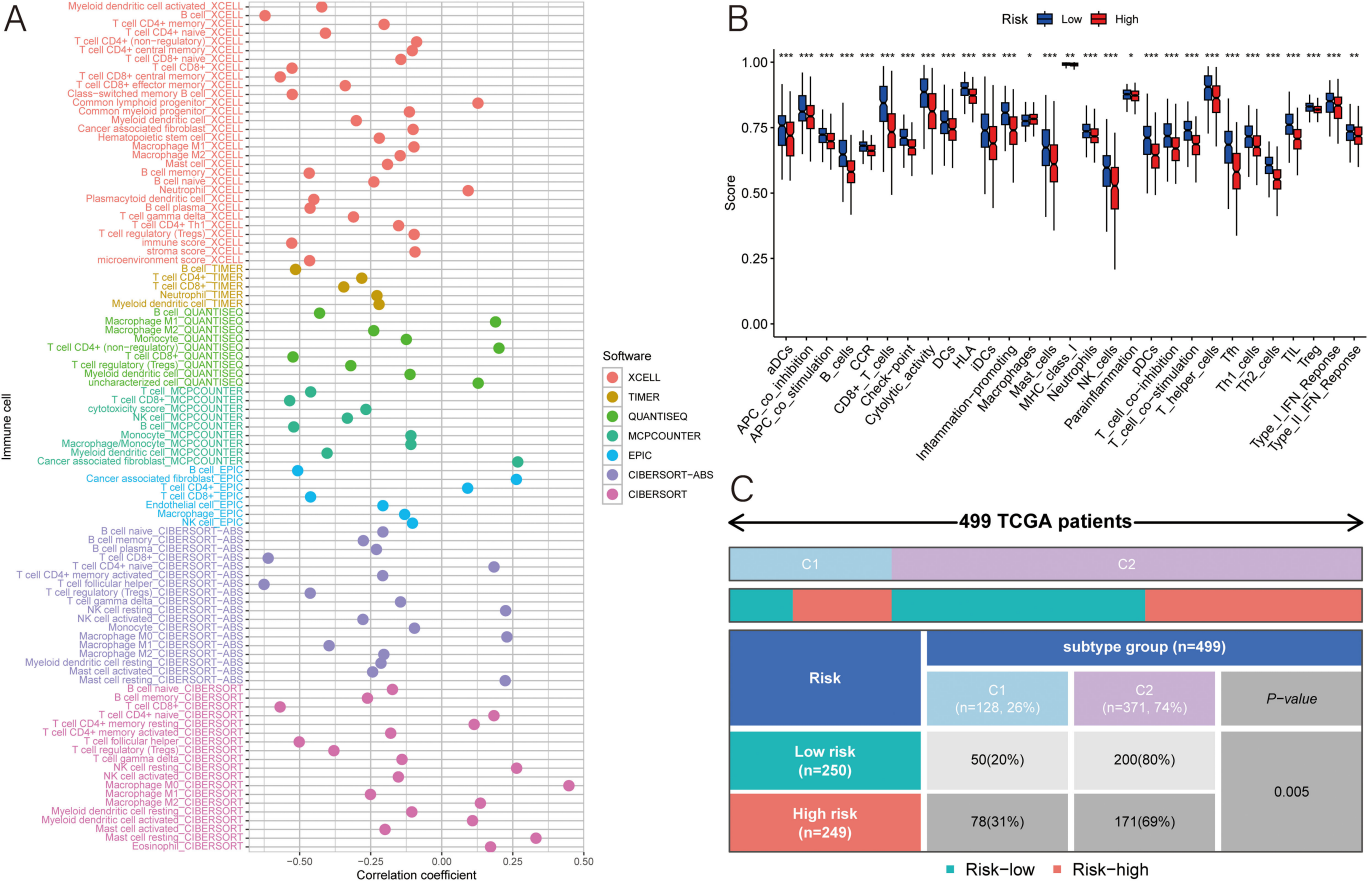
Immune analyses. **(A)** Multiple algorithms were applied to assess the relationship between MLDPM and immune cell subtypes. **(B)** Immune cell populations and functions were determined using ssGSEA. **(C)** chi-square test of different immune subtypes in two groups. ***p < 0.001; **p < 0.01; *p < 0.05.

A correlation analysis with seven different algorithms revealed a strong connection between the MLDPM and the expression levels of various immune cell populations. In the bubble chart, most immune cells had a higher expression in the low-risk group, and only CD4+ T cell and macrophage expressed higher in the high-risk group (**Figure 6A**). To further discover the immune microenvironment, we estimated the immune function by “ssGSEA” package. The result supported that only macrophages have a higher enrichment in the high-risk tumor tissue (**Figure 6B**). And HNSCC symbols were divided into different immune subtypes which are based on the consensus clustering of cell type proportions (C1-C6). Through Chi-squared test, it was discovered that there were statistical differences between groups in immune subtypes. The Immune subtype C2 (INF-gamma dominant) was more in the high-risk group and C1 (wound healing) was more in the low-risk group (**Figure 6C**).

### Predictive value of drug sensitivity

**Figure 7:**
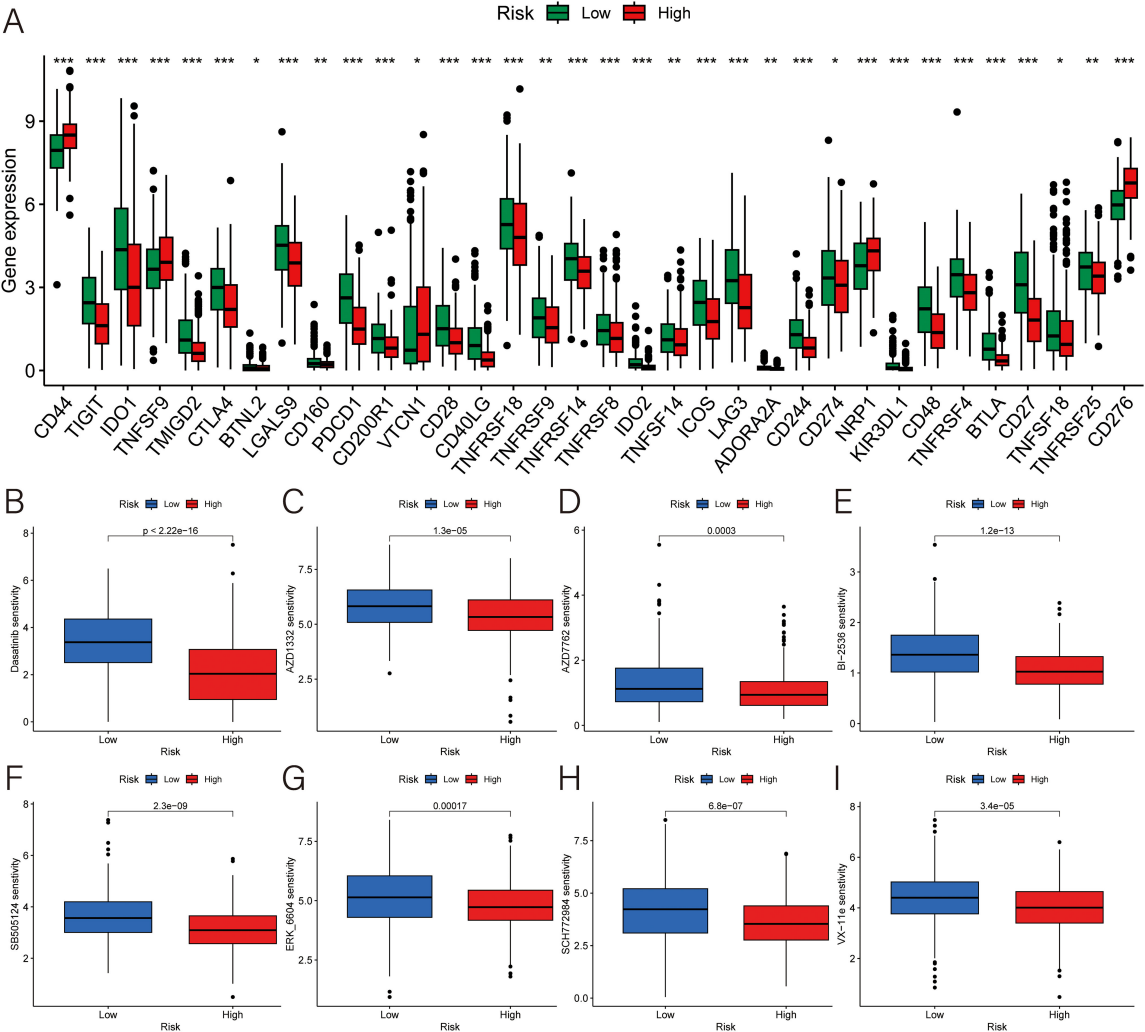
Exploration for the potential therapeutic strategies. **(A)** Expression of immune checkpoint genes. **(B–I)** The difference of the IC50 of **(B)** dasatinib for src/bcr-abl inhibitor, **(C)** AZD1332 for TRKA antagonist, **(D)** AZD7762 for checkpoint kinase inhibitor, **(E)** BI-2536 for PLK1 inhibitor, **(F)** SB505124 for ALK inhibitor, **(G)** ERK-6604, **(H)** SCH772984, and **(I)** VX-11e for ERK inhibitor. ***p < 0.001; **p < 0.01; *p < 0.05.

For evaluate the responsiveness to immunotherapy more accurately, the study delved into 47 immune checkpoint molecules of two groups, encompassing the B7/CD28 family, which regulates T-cell activation, and the TNF superfamily, known for their involvement in immune cell signaling. The result illustrated that a higher risk score was associated with lower expression of TIGIT, IDO1, TMIGD2, CTLA4, BTNL2, LGALS9, CD160, PDCD1, CD200R1, CD28, CD40LG, TNFRSF18, TNFRSF9, TNFRSF14, TNFRSF8, IDO2, TNFSF14, ICOS, LAG3, ADORA2A, CD244, CD274, KIR3DL1, CD48, TNFRSF4, BTLA, CD27, TNFSF18, TNFRSF25; and higher expression of CD44, TNFSF9, VTCN1, NRP1 and CD276 (**Figure 7A**). Therefore, patients’ risk score can play a role in the selection of potential checkpoint agonist. In the drug sensitivity analyses, high-risk group was more resistant to the immunotherapy. There were only 8 medicines had a better performance in the high-risk group (**Figures 7B–I**).

## Discussion

The complex and heterogeneous nature of HNSCCs poses significant challenges for current diagnostic and prognostic tools, leading to many misdiagnoses, undertreatment, or overtreatment (14). Given its unique anatomical location and the absence of effective early detection screening methods, HNSCC are often unresectable or diagnosed with early metastasis (15, 16). Immunotherapy has emerged as an essential component of treatment (14, 16). however, suitable treatment is contingent on accurate diagnosis. Consequently, there is an urgent need to develop a more precise method to provide guidelines for clinicians. The selection of appropriate algorithms and determining the optimal one require careful consideration, as individual preferences often dominate research choices. Therefore, establishing reliable prognostic biomarkers using optimal integrative machine learning algorithms is crucial.

In this study, we selected 18 remarkably different genes as a prognostic feature. For construct a MLDPM, TCGA database is used to train and GSE65858 is utilized to test via 101 machine learning algorithms (17). The risk assessment based on the validated model revealed that individuals classified as low-risk had significantly prolonged survival and superior prognosis. The ROC curve validated the signature’s reliability and stability, showcasing its commendable predictive accuracy. Notably, stage, age, and the risk score emerged as significant predictors, affirming the signature’s standalone value in forecasting HNSCC outcomes. Further analysis through ROC and C-index confirmed the exceptional performance of the MLDPM across various cohorts, suggesting its strong potential for practical implementation in clinical scenarios. Consequently, our findings suggest that MLDPM can effectively contribute to the assessment of prognosis for Head and Neck Squamous Cell Carcinomas in real-world medical settings, providing valuable guidance for treatment planning and patient management.

By compare differences between groups, we found that the low-risk group had lower TIDE scores and higher estimate scores. However, the difference in TMB between the two groups was minimal. GSEA enrichment analysis and immune function analysis indicate the immunological silence of the high-risk group (14). In the low-risk group, the tumor tissue has a bigger proportion immune subtype C1 (wound healing) than the high-risk group. On the contrast, immune subtype C2 (IFN-γDominant) has accounted for 80 percent of the tissue in the high-risk group. The immune response may lose control of tumors comprising C2, or tumors in C2 are those that have already been remodeled by the existing robust Type I infiltrate and have escaped immune recognition (18). The result indicates that HNSCC is a cancer which have adequate blood supply (18). Based on the analysis in the tumor immune microenvironment, fibroblasts and CD4+ T cell, especially naive CD4+ T cells, showed their positive relationship with the risk score. It was already well known that CD4+ T cell plays a vital role in anti-tumor effector cells. So high naive or resting T cells could be a response of high malignancy. Tumors might suppresses T cells to keep them rest or naive (19). It not only reduced the amount and function of CD8+ T cells and cytotoxic T lymphocytes, but also marred the immune function of CD4+ T cells (19, 20). Enough nutrition and silently immune response may be the reasons that high-risk patients have shorter survival time.

Immunotherapy holds immense promise for HNSCC therapy, yet its efficacy remains limited, with a reported response rate hovering around 20% or lower. Currently, only PD1 and PD-L1 have been validated as predictive biomarkers of immune checkpoint inhibitor response in HNSCC (21, 22). While bevacizumab, a monoclonal antibody targeting VEGF, did not improve OS, it did improve the response rate and progression-free survival, albeit with increased toxicities (23). This study marks the beginning of immunotherapy in NHSCC, aiming to investigate novel approaches to enhance the immunotherapy response rate and provide new possibilities for drug development.

Through drug sensitivity analysis, we found that 8 chemotherapeutics were more sensitive for the high-risk group. Three of them, ERK6604, SCH772984, as well as VX-11e, are extracellular regulated protein kinase (ERK) inhibitors. Raf-MEK-ERK pathway governs varied biological activities, including cell proliferation, migration, differentiation, and apoptosis (24). ERK 1/2 promotes cancer cell proliferation via angiogenesis, cell cycle entry, and enhanced survival (25). Therefore, ERK inhibitors can be a key of HNSCC treatment. And there are 102 chemotherapeutics were more suitable for the low-risk group, such as Afatinib, Afuresertib, Alisertib and so on. Those FDA‐approved drugs may be potential candidate agents for HNSCC. Upregulated genes like INHBA, PSMD2 and OLR1 play a crucial role in breast cancer (BC) which may bring us some similarities between HNSCC and BC (26–29). The high expression of CD44, TNFSF9, VTCN1, NR1, and CD276 in the high-risk group may show us specific molecular mechanism.

By using this model, we can divide HNSCC patients into different risk groups with their risk scores. And their degree of malignancy and survival time can be prognosticated by doctors to some extent. This is benefit for patients and doctors to take proactive cure measures or conservative treatment. From the study, Raf-MEK-ERK pathway might be an effective target for the research of HNSCC treatment. And it could reveal some physiological mechanisms in the cancer cell to help scientists prevent the progression and proliferation. And for immunotherapy, there are some possible drug and potential targets to provide new research directions for HNSCC treatment.

In conclusion, the present study highlights the transformative potential of the MLDPM in the clinical management of HNSCC patients. By offering a more nuanced understanding of patients’ immunophenotypes and delving deeper into the immune molecular mechanisms of the disease, MLDPM can help stratify patients more accurately, predicting response to immunotherapy and guiding personalized treatment plans. This novel approach has the potential to significantly improve patient outcomes and bridge the gap in immunotherapy response rates, making it a valuable addition to current diagnostic and therapeutic strategies in HNSCC care. While MLDPM is a promising comprehensively prognostic model, this study has some limitations. Firstly, the data of our study is limited. It only contained 270 samples from GEO database and 555 samples from TCGA-HNSC. And statistical bias, personal equation and lack of data might have bad influence on the result due to the different origins of profiles. There is a need to further validation on other data sets and larger clinical samples. Secondly, this model was not verified by clinical samples and experiment. There are certain limitations to clinical application. Thirdly, the study only explored the difference of drug sensitivity between two risk groups. But the effectiveness of drugs did not be validated. Finally, HNSCC is a general term of several cancers (22, 30, 31). Detailed analysis is needed to perform in the subtypes (22, 30, 31).

## Conclusion

In conclusion, we conducted a MLDPM through 101 algorithms combinations. In addition to the expression of immune genes, immune cells and checkpoints were explored between the high and low risk groups of the MLDPM. Meanwhile, the prediction and selection of individual and personalized immunotherapeutic can be facilitated by the MLDPM.

## Data Availability

The original contributions presented in the study are included in the article/**Supplementary Material**. Further inquiries can be directed to the corresponding authors.
